# Chronic Sleep Restriction in Developing Male Mice Results in Long Lasting Behavior Impairments

**DOI:** 10.3389/fnbeh.2019.00090

**Published:** 2019-05-03

**Authors:** Rachel Michelle Saré, Alex Song, Merlin Levine, Abigail Lemons, Inna Loutaev, Carrie Sheeler, Christine Hildreth, Angel Mfon, Spencer Cooke, Carolyn Beebe Smith

**Affiliations:** Section on Neuroadaptation and Protein Metabolism, Department of Health and Human Services, National Institute of Mental Health, National Institutes of Health, Bethesda, MD, United States

**Keywords:** chronic sleep-restriction, gentle handling, social behavior, autism, mammalian target of rapamycin

## Abstract

Sleep abnormalities are prevalent in autism spectrum disorders (ASD). Moreover, the severity of ASD symptoms are correlated with the degree of disturbed sleep. We asked if disturbed sleep during brain development itself could lead to ASD-like symptoms, particularly behavioral manifestations. We reasoned that sleep is known to be important for normal brain development and plasticity, so disrupted sleep during development might result in changes that contribute to behavioral impairments associated with ASD. We sleep-restricted C57BL/6J male mice [beginning at postnatal day 5 (P5) and continuing through P52] 3 h per day by means of gentle handling and compared the data with a stress group (handled every 15 min during the 3-h period) and a control group (no additional handling). From P42–P52, we assessed the behavioral effects of sleep-restriction in this pre-recovery phase. Then, we allowed the mice to recover for 4 weeks and tested behavior once again. Compared to the control group, we found that sleep restricted-mice had long-lasting hypoactivity, and impaired social behavior; repetitive behavior was unaffected. These behavior changes were accompanied by an increase in the downstream signaling products of the mammalian target of rapamycin pathway. These data affirm the importance of undisturbed sleep during development and show that, at least in this model, sleep-restriction can play a causative role in the development of behavioral abnormalities. Assessing and treating sleep abnormalities in ASD may be important in alleviating some of the symptoms.

## Introduction

Sleep is important for brain development and plasticity (Peirano and Algarin, 2007). In keeping with this, sleep deficiencies are prevalent in neurodevelopmental disorders such as ASDs (Picchioni et al., 2014). In addition, these sleep problems may be more prevalent than realized. In a population of patients with high-functioning ASD, even among those who did not complain of sleep problems, ASD patients had a significantly increased sleep latency and decreased sleep efficiency compared to controls (Limoges et al., 2013). Moreover, the prevalence of sleep disorders in ASD is correlated with the severity of the ASD-specific behaviors including social impairments (Schreck et al., 2004; Veatch et al., 2017). Parent-reported total sleep duration was most strongly correlated with social behavior abnormalities (Veatch et al., 2017). Sleep loss is also linked to ASD on a molecular level. Sleep loss, either acute or chronic, can lead to many molecular changes that can alter plasticity and are linked to ASD and other neurodevelopmental disorders (Picchioni et al., 2014).

In C57BL/6J mice, we have previously shown that chronic sleep-restriction resulted in long-lasting impairments in activity, sleep, and social behavior (Sare et al., 2015). The changes in social behavior were sex-specific, showing that sleep-restricted females had increased sociability while this was not true of males. The data for the male mice were underpowered to be definitive but did hint at possible changes. Given the prevalence of ASD in males, we have repeated these studies on a larger cohort of male mice. Additionally, we have added a group that was stressed but not sleep-restricted to determine if behavioral changes were due to stress *per se*.

We performed chronic sleep-restriction for 3 h per day throughout development. Immediately after sleep-restriction, we performed a battery of behavior tests to examine hyperactivity, anxiety, repetitive behavior, and social behavior. Then, we allowed the mice to recover for 4 weeks, during which time, we monitored sleep. After the 4 weeks of recovery, we repeated the battery of behavior tests. Our results indicate that even after recovery sleep, sleep-restricted male mice had reduced activity, and reduced preference for social novelty.

These results indicate that sleep-restriction during development in males can lead to the development of ASD-like behaviors. These results are important in highlighting the role of sleep during development in long-term behavior and the possible role of sleep disturbance in the pathophysiology of ASD. Sleep disturbances should be considered in the primary care for the treatment of ASD.

## Materials and Methods

### Animals

Mice on a C57Bl/6J background were generated in house from our Fragile X colony (*C57BL/6J-Fmr1*^tm1Cgr^) (we used the littermate controls of the *Fmr1* KO colony produced from control males and heterozygous females). New C57BL/6J mice from Jackson labs were periodically crossed back into the colony. Once a female gave birth, the dam and her pups were separated from the sire in the cage. At 10 days of age, ear punches were taken for later identification and determination of genotypes as previously described (Qin et al., 2005). Pups were weaned at 21 days of age. Mice were group housed in a climate-controlled facility (maintained between 70–75°C and between 20–30% humidity) on a standard 12:12 light:dark cycle (lights on at 6AM). Food and water were available *ad libitum*. All procedures were performed in accordance with the National Institutes of Health Guidelines on the Care and Use of Animals and approved by the National Institute of Mental Health Animal Care and Use Committee.

### Sleep-Restriction

**FIGURE 1:**
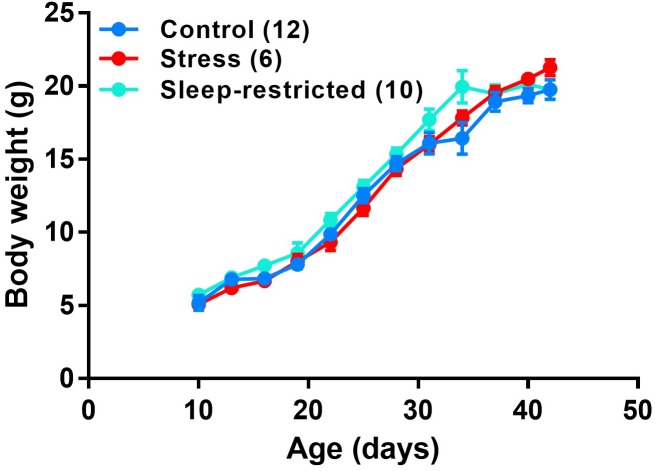
Growth curves of male C57BL/6J mice under three conditions: control, stress, and sleep-restriction. Each point represents the mean ± SEM for the number of mice indicated in parentheses.

Litters of animals were randomly assigned to one of three groups: (1) control, (2) stress, or (3) sleep-restriction. The stress and sleep-restricted groups were transferred to a different room in the animal facility; controls remained in the housing room. Each manipulation was performed on the entire litter. For the sleep-restriction group, sleep-restriction was initiated at P5, 3 h per day from 11:00 AM to 2:00 PM, by gentle handling as previously described (Sare et al., 2015; Lemons et al., 2018). Neonatal animals (before P10) had to be prodded almost constantly to keep them awake. After P10, they required less prodding to stay awake, but the need for prodding increased as sleep-restriction progressed. If an animal was suspected to enter sleep, the animal was gently prodded until movement was observed to ensure the animal was awake. For the 3-h period, mice in the stress group were prodded with the paint brush once every 15 min regardless of their sleep state. Every 3 days, beginning at P10, mice were weighed to assess growth. Sleep-restriction did not impair growth (Figure 1).

### Behavior Testing

Behavior testing was initiated at P42. All animals in the behavior group underwent all behavior tests (Control *n* = 28, Stress *n* = 21, and Sleep Restriction *n* = 24), though certain data were not obtained for reasons listed below. Testing was performed in the following order: open field, marble burying, social behavior, and social transmission of food preference. Tests were separated by a 2-day gap, during which time, sleep-restriction was performed. Sleep-restriction was not performed on the day of testing. Testing was conducted between 11:00 AM and 2:00 PM. After the last behavior test, animals recovered for 4 weeks and were permitted to sleep *ad libitum*. During this time, we assessed sleep behavior. Following the recovery period, animals were retested with the same battery of behavioral tests with no additional sleep-restriction. The schedule of sleep-restriction and testing is presented in Table 1.

**Table 1:** Experiment schedule.

Age (days)	Procedure
5–52	Sleep restriction
42	Open field
45	Marble burying
48	Social behavior
50–52	Social transmission of food preference
52–84	Recovery sleep
74–77	Sleep testing
84	Open field
87	Marble burying
90	Social behavior
92–94	Social transmission of food preference
94	Harvest for western blotting


### Open Field

Open field testing was performed using the Coulbourn TruScan system (Coulbourn Instruments, Whitehall, PA, United States) as previously described (Sare et al., 2015). Animals were placed in the novel chamber (26 cm square box) for 30 min. Data were collected in six epochs, each lasting 5 min. Total horizontal distance traveled, and the ratio of center distance (more than 6.25 cm from the wall) to total distance were analyzed across the six epochs. Due to equipment failure, we lost data for three control, three stress, and one sleep restriction animal.

### Marble Burying

Marble burying was conducted as a measure of repetitive behaviors as previously described (Sare et al., 2015). Each animal was placed in a cage (standard housing cage: 31 cm × 16.5 cm) with thick hardwood bedding (4.5 cm in depth) containing a grid of 20 glass marbles (5 × 4) placed on the surface and mice were left to explore the cage for 30 min. Afterwards, the total number of marbles buried (>50% coverage) was determined.

### Social Behavior

Social behavior was conducted with an automated three chambered apparatus as previously described (Sare et al., 2016). There were three phases of the test, each lasting 5 min. (1) Habituation: animals were placed in the empty apparatus and allowed to habituate to all three chambers. If an animal spent more than 3 min in a chamber or avoided a chamber completely, it was excluded from the test. (2) Sociability: a novel age/sex-matched stranger mouse (also C57BL/6J) was placed in a sociability cup (Noldus, Leesburg, VA, United States) in either Chambers 1 or 3, and an empty sociability cup was placed into the opposite chamber. The test mouse was allowed to explore all three chambers for 5 min. (3) Social Novelty: a novel age/sex-matched stranger mouse was placed in the previously empty sociability cup. The test mouse was allowed to explore all three chambers for 5 min. During each phase, the time spent in each chamber was recorded. Sessions were video-recorded for later assessment of sniffing behavior. Sniffing was determined by automated software (TopScan) (Clever Systems, Reston, VA, United States) based on proximity of the nose to the cup (<20 mm). Different stranger mice were used for each time point (before and after recovery) of testing. We started with 28 control mice, though eight were excluded due to operator error, three due to equipment malfunction, and three due to a chamber preference during the habituation phase. We started with 21 stress mice, though four were excluded due to operator error, one due to equipment malfunction, and three due to a chamber preference during the habituation phase. Six additional mice were excluded from the sniffing time analysis (five due to missing files, and one due to operator error). We started with 24 sleep restriction mice, though four were excluded due to operator error, and three due to a chamber preference during the habituation phase. We excluded an additional mouse for sniffing analysis due to operator error.

### Social Transmission of Food Preference

One animal from each cage was randomly assigned to be the demonstrator mouse. The demonstrator mouse was separated from the cage mates (observer mice), singly housed, and food-deprived for 18 h. Then, he was given a novel food (randomly assigned) of either 2% cocoa or 1% cinnamon for 1 h. Then, the demonstrator interacted with its cage mates for 30 min. After this time, the cage mates were food deprived for 18 h. Then, the cage mates were singly housed for 1 h and given the choice between the two flavored foods (cocoa or cinnamon). The amount of each food consumed was recorded. Note, for the second round of testing, after the recovery sleep period, new flavors were used: 1% cloves and 1% onion. We do not present the results in this paper because, in our hands, control mice did not perform above chance. We present the procedure because it was part of the series of tests all mice in the study underwent.

### Sleep Testing

Animals were separated into individual standard mouse cages and placed in the activity monitoring system (CLAMS) (Columbus Instruments, Columbus, OH, United States) for 72 h. To allow for habituation to the single housing and monitoring system, only the last 48 h-period was analyzed (Sare et al., 2018). Activity was recorded in 10 s epochs. Four consecutive epochs of inactivity were recorded as sleep. The total time asleep in both the active and the inactive phases of the circadian cycle was recorded. Sleep duration is reported as a percentage of the 12-h recording period in either the active or the inactive phase. Due to equipment failure, we lost data for four control, two stress, and one sleep restriction animal.

### Western Blotting

Mice were decapitated between 2 and 3 PM, brain regions were rapidly dissected, placed in Precellys lysis tubes (Bertin Corporation, Rockville, MD, United States), and stored at -80°C.

Tissue was thawed at 4°C, homogenized with the Precellys Homogenizer in 5% (weight/volume) Tissue Protein Extraction Regent (Thermo Scientific, Waltham, MA, United States) with 1% Halt Protease and Phosphatase Inhibitor Cocktail (Thermo Scientific) and 1% 0.5 M ethylenediaminetetraacetic acid (EDTA) solution (Thermo Scientific). Protein concentrations were determined with a Pierce BCA Protein Assay Kit (Thermo Scientific). We used the Bio-Rad mini-protein stain-free gel technology and the Trans-Blot Turbo system for subsequent Western blot analysis (Bio-Rad, Hercules, CA, United States) (Sare et al., 2017).

Primary antibodies were diluted at 1:1,000: p-4EBP1 (Eukaryotic translation initiation factor 4E-binding protein 1) (Cell Signaling 9455), p-AKT (Protein kinase B) (Cell Signaling 4060), p-AMPK (AMP-activated protein kinase) (Cell Signaling 2535), CLOCK (Circadian Locomotor Output Cycles Kaput) (Bethyl A302-618A), p-CREB (cAMP response element-binding protein) (Cell Signaling 9198), p-eIF2α (Eukaryotic translation initiation factor 2α) (Cell Signaling 3398), p-ERK (Extracellular regulated kinase) (Cell Signaling 3370), p-GSK3 (Glycogen synthase kinase 3) (Cell Signaling 9931), Iba1 (ionized calcium-binding adapter molecule 1) (Abcam AB48004), p-JNK (C-Jun N-terminal kinase) (Cell Signaling 9251), LC3 (microtubule-associated protein 1A/1B light chain 3) (Abgent AP1802a), MBP (myelin basic protein) (Proteintech 10458-1-AP), p-mTOR (mammalian target of rapamycin) (Cell Signaling 5526), p-p70S6K (ribosomal protein S6 kinase) (Cell Signaling 9234), p-S6 (ribosomal protein S6) 235/236 (Cell Signaling 2211), and p-S6 240/244 (Cell Signaling 2215) and incubated overnight with the membrane at 4°C.

### Corticosterone Analysis

Animals were sleep-restricted in the manner described above from P5–P9 or from P5–P42. Immediately following sleep-restriction (between 2:00 and 3:00 PM), serum was collected from decapitated animals, and stored at -80°C.

Serum was thawed at 4°C, diluted 1:200 with the diluent provided in the ImmuChem ^125^I Corticosterone Radioimmunoassay Kit (MP Biomedicals, Solon, OH, United States). The assay was conducted per kit instructions and corticosterone levels were calculated based on the acquired standard curve.

### Statistical Analysis

Data are represented as means ± SEM. Data were analyzed by means of a three-way mixed-model repeated measures ANOVA with condition (control, stress, or sleep restriction) as between subjects’ variables and age (pre or post recovery), and epoch (open field), chamber (social behavior), or phase (sleep) as within subjects’ variables (SPSS, IBM, Armonk, NY, United States). Corticosterone and marble burying were analyzed by means of a two-way ANOVA. Western blot results were analyzed by means of one-way ANOVA. Effects with *p* < 0.05 were considered statistically significant and are indicated with a “^∗^”, though values 0.05 < *p* < 0.10 are reported here with a corresponding “∼.” Tables reporting *F*-values and corresponding *p*-values for interactions and main effects are presented for all the ANOVA data (Tables 2–4).

**Table 2:** Repeated measures ANOVA results.

Test	Interaction	Main effect	*F*_(df,error)_ value	*P*-value
Corticosterone	Age × condition		*F*_(2,45)_ = 1.980	0.150
		Age	*F*_(1,45)_ = 32.154	<0.001*
		Condition	*F*_(2,45)_ = 1.513	0.231
Sleep				
Total sleep time	Condition × phase		*F*_(2,62)_ = 3.008	0.057∼
		Condition	*F*_(2,63)_ = 0.424	0.656
		Phase	*F*_(1,63)_ = 1285.364	<0.001*
Open field				
Total distance moved	Age × condition × epoch		*F*_(8,250)_ = 0.839	0.568
	Condition × epoch		*F*_(7,209)_ = 0.439	0.869
	Age × epoch		*F*_(4,250)_ = 2.920	0.022*
	Age × condition		*F*_(2,63)_ = 0.582	0.562
		Age	*F*_(1,63)_ = 5.954	0.018*
		Condition	*F*_(2,63)_ = 7.560	0.001*
		Epoch	*F*_(3,209)_ = 116.606	<0.001*
Center/total ratio	Age × condition × epoch		*F*_(9,282)_ = 0.611	0.787
	Condition × epoch		*F*_(10,315)_ = 1.806	0.059∼
	Age × epoch		*F*_(4,282)_ = 0.532	0.733
	Age × condition		*F*_(2,63)_ = 2.782	0.070∼
		Age	*F*_(1,63)_ = 34.224	<0.001*
		Condition	*F*_(2,63)_ = 0.475	0.624
		Epoch	*F*_(5,315)_ = 7.577	<0.001*
Marble burying				
Buried	Age × condition		*F*_(2,70)_ = 2.846	0.065∼
		Age	*F*_(1,70)_ = 27.739	<0.001*
		Condition	*F*_(2,70)_ = 0.156	0.856
Sociability				
Time in chamber	Age × condition × chamber		*F*_(2,42)_ = 4.229	0.021*
	Condition × chamber		*F*_(2,42)_ = 6.007	0.005*
	Age × chamber		*F*_(1,42)_ = 0.886	0.352
	Age × condition		*F*_(2,42)_ = 0.062	0.940
		Age	*F*_(1,42)_ = 0.994	0.324
		Condition	*F*_(2,42)_ = 4.210	0.022*
		Chamber	*F*_(1,42)_ = 73.338	<0.001*
Sniffing	Age × condition × chamber		*F*_(2,35)_ = 0.297	0.745
	Condition × chamber		*F*_(2,35)_ = 0.342	0.713
	Age × chamber		*F*_(1,35)_ = 0.009	0.924
	Age × condition		*F*_(2,35)_ = 0.288	0.751
		Age	*F*_(1,35)_ = 5.712	0.022*
		Condition	*F*_(2,35)_ = 0.188	0.829
		Chamber	*F*_(1,35)_ = 56.838	<0.001*
Social novelty				
Time in chamber	Age × condition × chamber		*F*_(2,42)_ = 3.093	0.056∼
	Condition × chamber		*F*_(2,42)_ = 0.298	0.744
	Age × chamber		*F*_(1,42)_ = 0.013	0.911
	Age × condition		*F*_(2,42)_ = 1.472	0.241
		Age	*F*_(1,42)_ = 2.297	0.137
		Condition	*F*_(2,42)_ = 3.510	0.039*
		Chamber	*F*_(1,42)_ = 0.631	0.432
Sniffing	Age × condition × chamber		*F*_(2,35)_ = 1.500	0.237
	Condition × chamber		*F*_(2,35)_ = 3.031	0.061∼
	Agee × chamber		*F*_(1,35)_ = 1.580	0.217
	Age × condition		*F*_(2,35)_ = 1.087	0.348
		Age	*F*_(1,35)_ = 1.011	0.322
		Condition	*F*_(2,35)_ = 0.415	0.664
		Chamber	*F*_(1,35)_ = 2.453	0.126


## Results

### Corticosterone Levels

**FIGURE 2:**
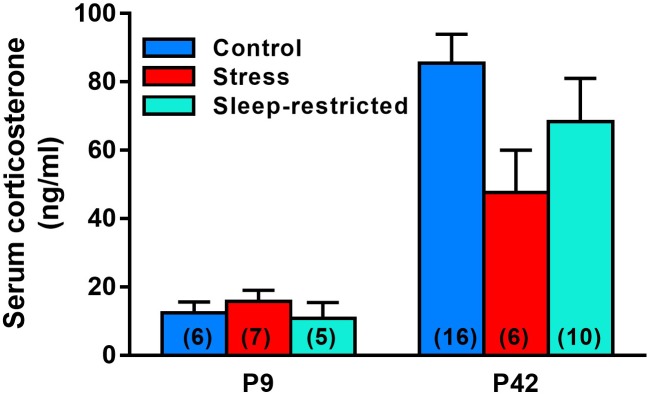
Serum corticosterone concentrations at P9 and P42 after control, stress, and sleep-restriction commencing at P5. Bars represent mean ± SEM for the number of mice indicated on the figure. Results of ANOVA are given in Table 2.

As a measure of stress, we determined serum levels of corticosterone in mice subjected to the three treatments described (control, stress, and sleep-restriction). Trunk blood was collected at two different time points, P9 and at P42 (Figure 2). We did not find a statistically significant interaction between age and condition, but the main effect of age was highly significant (Table 2). In control mice, serum concentrations of corticosterone at P9 were 15% of concentrations at P42. The main effect of condition was not statistically significant, but at P42 mean serum corticosterone concentration was 44% lower in the stress group and 20% lower in the sleep-restricted group compared to controls (Figure 2). In the animals at P9, mean values of serum corticosterone concentrations were similar in all three groups. These results indicate that a stress response to sleep-restriction is likely not responsible for the effects on behavior.

### Sleep Duration

**FIGURE 3:**
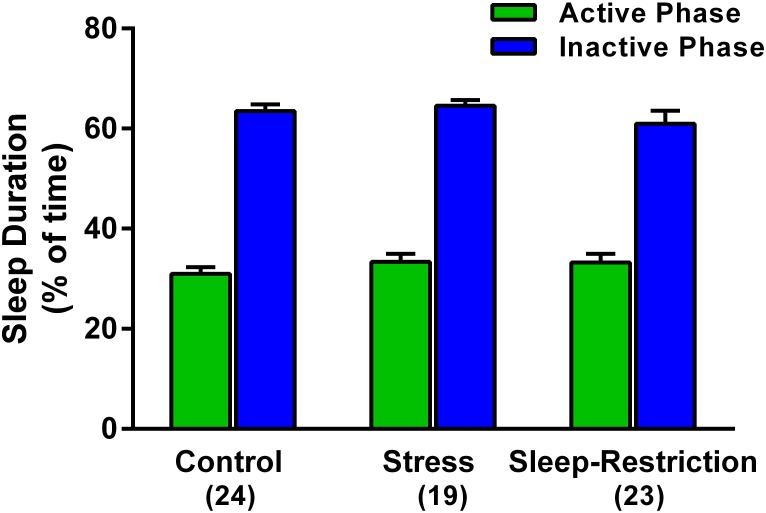
Effects of stress and sleep-restriction conditions on sleep duration in the active and inactive phases of the circadian cycle measured from P74–P77. Bars represent mean ± SEM for the number of mice indicated. Results of ANOVA are given in Table 2.

We asked if stress or sleep-restriction from P5–P52 influenced sleep duration after 3 weeks of recovery sleep. We used home-cage activity-based monitoring to record total sleep duration during the active and inactive phases of the circadian cycle from P74–P77. There was a near significant condition × phase interaction *p* = 0.057 (Table 2). However, there were no significant pair-wise differences in sleep duration between the groups in either of the phases (Figure 3).

### Activity Response to a Novel Environment

**FIGURE 4:**
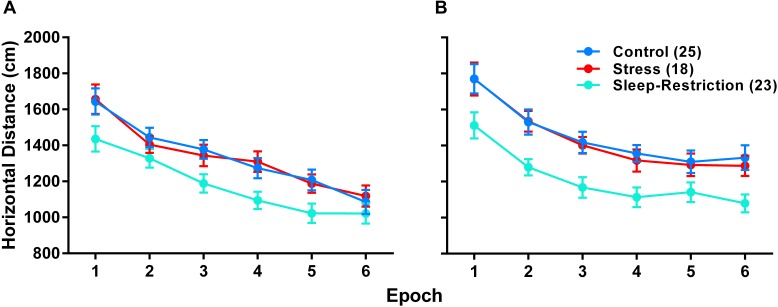
Sleep-restriction resulted in hypoactivity both pre- (P42) **(A)** and post-recovery (P84). **(B)** The main effect of condition was statistically significant (*p* < 0.001), indicating that, regardless of epoch or age, sleep-restriction resulted in decreased activity levels compared to control and stress groups. Each point represents the mean ± SEM for the number of mice indicated in parentheses.

To examine the effects of stress and sleep-restriction on the response to a novel environment, we measured horizonal distance traveled in an open field at two time points: P42 (during sleep-restriction) and P84 (following recovery sleep) (Figure 4). We found a statistically significant age × epoch interaction (*p* = 0.022) (Table 2) indicating a change in habituation to the novel environment across the two testing points regardless of condition. Older mice appear to have habituated more rapidly than younger animals. We also found a statistically significant main effect of age (*p* = 0.018) indicating that mice regardless of condition were more active at P84 compared with P42. The main effect of condition (*p* < 0.001) (Table 2) was also statistically significant indicating that, at both time points, sleep-restricted mice were less active compared to the control and stress groups. Control and stress groups showed similar activity levels at both time points.

### Anxiety-Like Behavior

**FIGURE 5:**
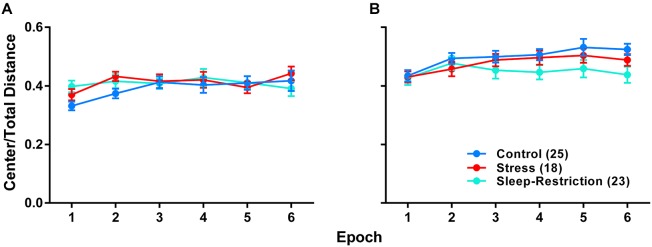
Effects of stress and sleep-restriction on anxiety-like behavior at P42 **(A)** and following recovery at P84 **(B)**. Results of ANOVA are given in Table 2. Both the condition × epoch (*p* = 0.059) and age × condition (*p* = 0.070) interactions approached statistical significance suggesting that, regardless of age, stress and sleep-restriction changed the habituation response to the novel environment. Each point represents the mean ± SEM for the number of mice indicated in parentheses.

We analyzed the ratio of distance traveled in the center of the open field to the total distance traveled as an index of anxiety-like behavior (Figure 5). Our goal was to see if chronic sleep-restriction had any acute (P42) or long-lasting (P84) effects on anxiety. We found a near significant age × condition interaction (*p* = 0.070) (Table 2), though *post hoc* tests did not reveal significant differences between conditions at the two ages. For all three conditions regardless of epoch, ratios were higher at P84 (*p* < 0.05), suggesting that anxiety decreased with age. We also found a near significant condition × epoch interaction (*p* = 0.059) (Table 2). Regardless of age, in epoch 6, the center to total distance ratio tended to be lower in sleep-restricted mice compared to controls (*p* = 0.084). At P84, the sleep-restricted group showed very little habituation to the open field in contrast to either control or stress groups (Figure 5).

### Marble Burying

**FIGURE 6:**
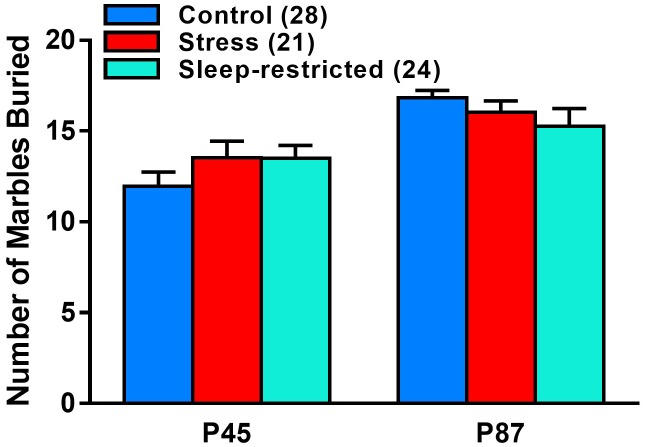
Effects of stress and sleep-restriction on repetitive behavior at P45 and P87. Results of ANOVA are given in Table 2. The age × condition interaction approached statistical significance (*p* = 0.065) suggesting that control and stress groups buried more marbles at P87 than at P45, but this behavior was not increased in the older sleep-restricted mice. Each bar is the mean ± SEM for the number of mice indicated in parentheses.

We measured marble burying at P45 and P87 to ascertain the effects of sleep-restriction on repetitive behavior (Figure 6). We found a near significant age × condition interaction (*p* = 0.065) (Table 2). *Post hoc* tests indicated no significant differences between conditions pre-and post-recovery. The effect of age on marble burying was statistically significant for both control (*p* < 0.001) and stress (*p* = 0.021) groups; both groups buried more marbles at P87 compared with P45. This was not the case for the sleep-restricted group, in which this repetitive behavior did not change significantly over time.

### Social Behavior

**FIGURE 7:**
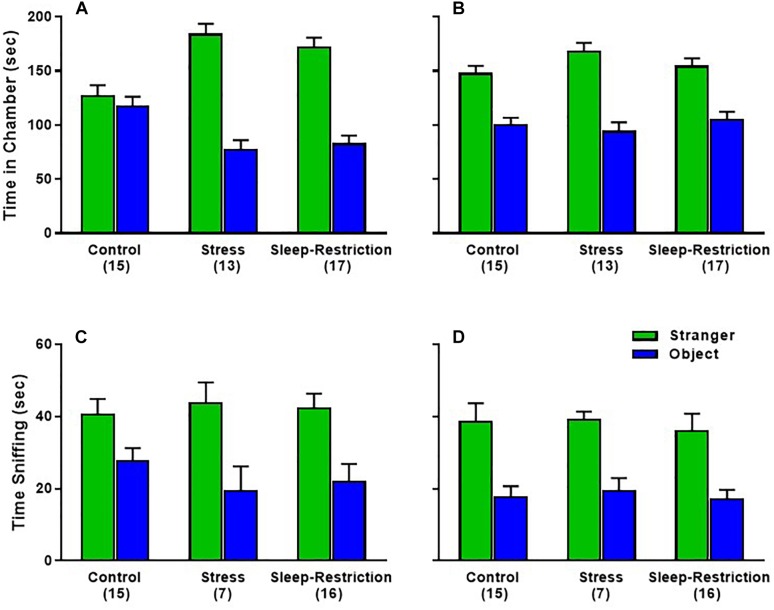
Effects of sleep-restriction on sociability measured at P48 **(A,C)** and at P90 **(B,D)** after recovery. Results of ANOVA are given in Table 2. **(A,B)** Time in Chamber: there was a significant age × condition × chamber interaction (*p* = 0.021). *Post hoc t*-tests indicate that only control animals at P48 did not have a significant preference for time in the chamber with the stranger mouse compared with the object. All other groups were statistically significant (*p* < 0.003). **(C,D)** Sniffing time: there were no statistically significant effects regarding condition. The main effect of chamber (*p* < 0.001) indicates that all mice, regardless of condition or age, showed a clear preference for the stranger mouse compared with the object. The main effect of age (*p* < 0.05) indicates that regardless of condition or chamber, mice sniffed less at P90 compared with P48. Ear bar is the mean ± SEM for the number of mice indicated in parentheses.

To examine social behavior, we tested the three groups of mice in the 3-chambered apparatus at P48 (during sleep restriction) and P90 (after recovery sleep). For the sociability phase, time spent in each chamber, the age × condition × chamber interaction was statistically significant (*p* = 0.021) (Table 2). *Post hoc* *t*-tests indicated that both the stress and sleep-restricted mice at P48 spent more time in the chamber with the stranger mouse compared with the chamber with only the object (*p* < 0.003) (Figure 7A,B). Control mice at P48 did not show this preference. All mice at P90 did show this preference. Because sleep-restricted mice were hypoactive in the open field, we were concerned that these mice may have spent more time in the center chamber, but this was not the case (Supplementary Figures S1A,B). All three groups of mice moved around all three chambers. We also analyzed time spent sniffing the stranger mouse and the novel object (Figure 7C,D). The main effect of age was statistically significant (*p* = 0.022) (Table 2), indicating that all mice, regardless of chamber or condition, spent less time sniffing at P90. The main effect of chamber was also statistically significant, indicating that all mice, regardless of age or condition, preferred interacting with the stranger mouse compared to the object (Figure 7C,D).

**FIGURE 8:**
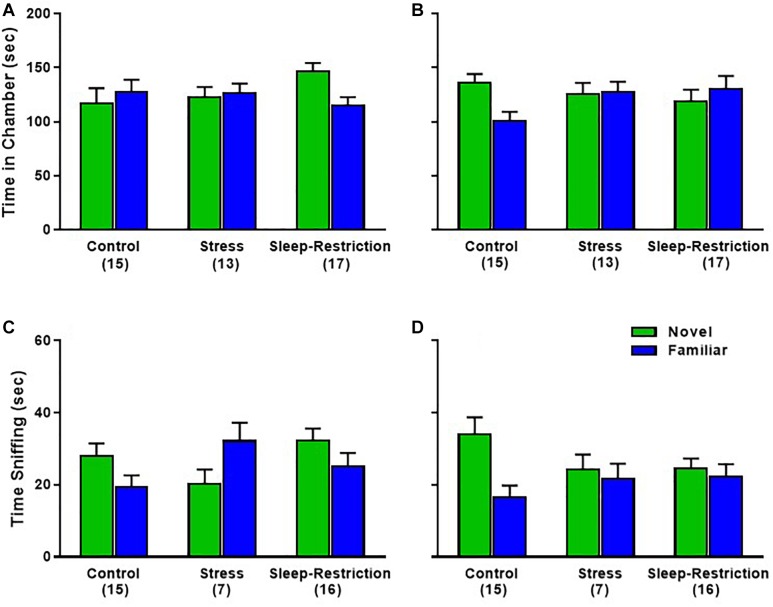
Effects of sleep-restriction on preference for social novelty measured at P48 **(A,C)** and at P90 **(B,D)** after recovery. Results of ANOVA are given in Table 2. **(A,B)** Time in Chamber: there was a nearly significant age × condition × chamber interaction (*p* = 0.056). We proceeded to probe for pairwise differences and found that sleep-restricted mice at P48 and control mice at P90 had a trend toward showing a preference for time spent in the chamber with the novel mouse compared to the familiar mouse (*p* = 0.091, *p* = 0.079, respectively). **(C,D)** Sniffing time: there was a near significant condition × chamber interaction (*p* = 0.061) suggesting that, regardless of age, control mice showed a significant preference for the novel mouse compared with the familiar mouse. Mice in the stress and sleep-restriction groups did not show this preference for social novelty. Each bar is the mean ± SEM for the number of mice indicated in parentheses.

In the preference for social novelty phase, time spent in each chamber, the age × condition × chamber interaction approached statistical significance (*p* = 0.056) (Table 2). We probed for pair-wise differences, but none of the comparisons were statistically significant (Figure 8A,B). Times spent in all three chambers, including the center chamber are also shown (Supplementary Figures S1C,D). Analysis of time spent sniffing during the preference for social novelty phase indicated a near significant condition × chamber interaction (*p* = 0.061) (Table 2). *Post hoc t*-tests showed that only control mice (regardless of age) had a statistically significant preference for the novel mouse compared to the familiar mouse (*p* = 0.003). Neither the stress nor sleep-restricted mice (regardless of age) had a preference (Figure 8C,D). These data suggest that both stress and sleep-restriction impaired the preference for social novelty in C57BL/6J mice.

### Molecular Changes

To assess the effects of stress or sleep-restriction on long-term molecular changes, we performed Western blotting in frontal cortex lysates using candidate proteins from mice at P94, following recovery sleep and behavior testing. We examined processes known to be altered in response to sleep deprivation that are implicated in brain plasticity (Picchioni et al., 2014). Specifically, we examined pathways involved in cell death (LC3, microtubule-associated protein 1A/1B light chain 3), cellular stress (JNK, c-Jun N-terminal kinases), circadian rhythm (CLOCK, circadian locomotor output cycles kaput), microglia (Iba1, allograft inflammatory factor 1), myelination (MBP, myelin basic protein), and transcription and translation (AKT, protein kinase B; CREB, cAMP response element-binding protein; eIF2α, eukaryotic initiation factor 2α; GSK3, glycogen synthase kinase 3; ERK, extracellular signal-regulated kinase, and mTOR, mammalian target of rapamycin).

**FIGURE 9:**
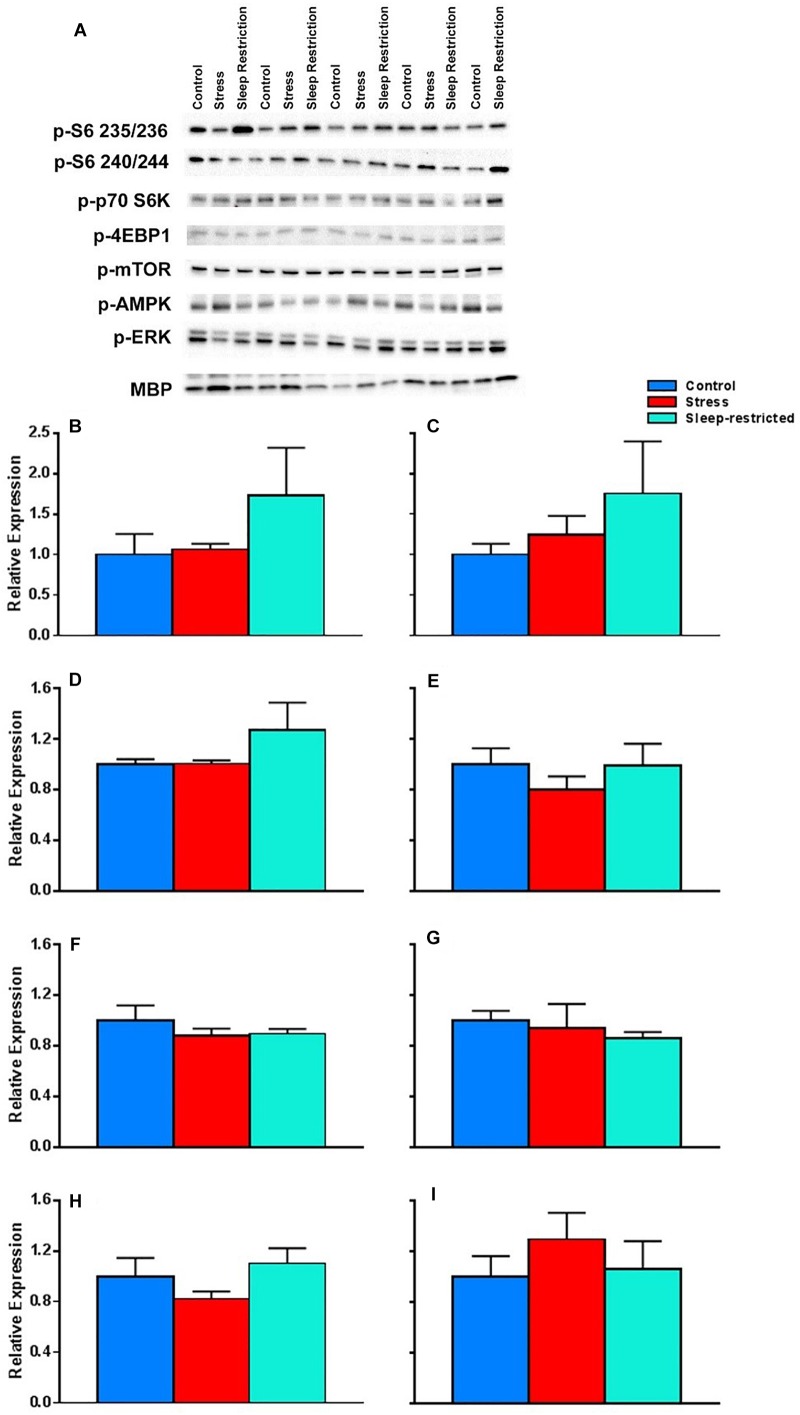
Effects on key proteins at P94. **(A)** Images of the blots. **(B–I)** Protein levels were normalized to controls for p-S6 235/236 **(B)**, p-S6 240/244 **(C)**, p-p70 S6K **(D)**, p-4EBP1 **(E)**, p-mTOR **(F)**, p-AMPK **(G)**, p-ERK **(H)**, and MBP **(I)**. Each bar represents mean ± SEM for control group (*n* = 5), stress group (*n* = 4), and sleep-restricted group (*n* = 5). Results of ANOVA are presented in Table 3. We found no statistically significant effects of condition.

We did not see any condition differences in p-AKT, CLOCK, p-CREB, p-eIF2α, p-GSK3, p-JNK, Iba1, or LC3 (data not shown). We also analyzed p-ERK and MBP and did not find any significant effects (Figure 9A,H,I). We interrogated components of the mTOR pathway: p-S6 235/236 (ribosomal protein S6), p-S6 240/244, p-p70 S6K (ribosomal protein S6 kinase), p-4EBP1 (eukaryotic translation initiation factor 4E-binding protein 1), p-mTOR, and pAMPK (5′ AMP-activated protein kinase) (Figure 9A–G). We did not detect any statistically significant effects of condition (Table 3).

**Table 3:** ANOVA results for western blots.

Protein	Main effect	*F*_(df,error)_ value	*P*-value
p-S6 235/236	Condition	*F*_(2,11)_ = 1.059	0.380
p-S6 240/244	Condition	*F*_(2,11)_ = 0.856	0.451
p-p70 S6K	Condition	*F*_(2,11)_ = 1.253	0.324
p-4EBP1	Condition	*F*_(2,11)_ = 0.581	0.576
p-mTOR	Condition	*F*_(2,11)_ = 0.639	0.546
p-AMPK	Condition	*F*_(2,11)_ = 0.471	0.636
p-ERK	Condition	*F*_(2,11)_ = 1.530	0.259


**FIGURE 10:**
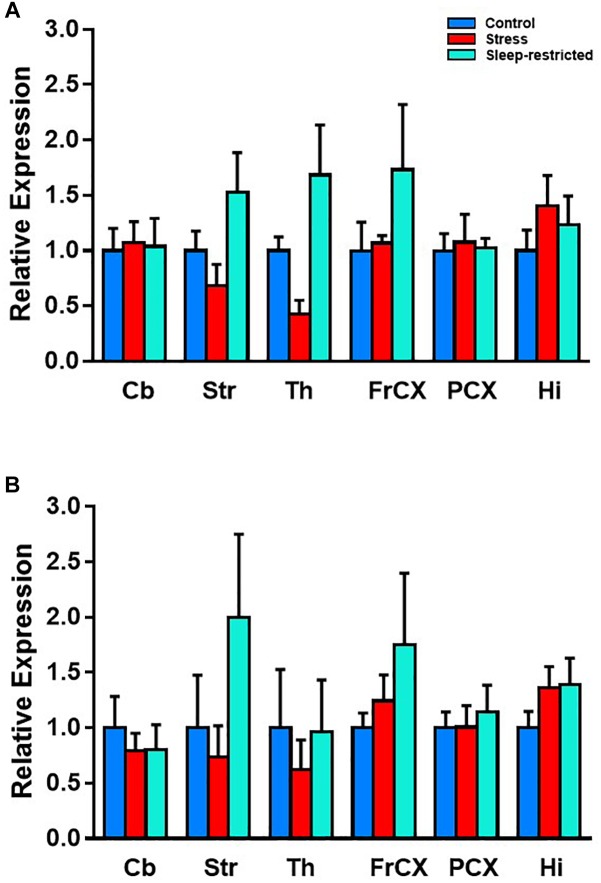
Effects on p-S6 protein expression in six brain regions at P94. We analyzed p-S6 235/236 **(A)** and p-S6 240/244 **(B)** in multiple brain regions. Protein levels were normalized to control values. Results of ANOVA are presented in Table 4. We found a statistically significant main effect of condition *p* = 0.038. This shows that, regardless of region or of phosphorylation site, p-S6 is increased following sleep restriction. Each bar represents mean ± SEM for control group (*n* = 5), stress group (*n* = 4), and sleep-restricted group (*n* = 5). Cb, cerebellum; Str, striatum; Th, thalamus; FrCX, frontal cortex; PCX, parietal cortex; and Hi, hippocampus.

Although we did not detect significant effects on pS6, we did note the large differences in mean values between control and sleep restriction. Therefore, we further probed S6 in different brain regions following stress and sleep-restriction. We analyzed both p-S6 235/236 and p-S6 240/244 in multiple brain regions frontal cortex, striatum, thalamus, parietal cortex, hippocampus, and cerebellum. We found a statistically significant main effect of condition (*p* = 0.038) (Table 4). This shows that, regardless of region or of phosphorylation site, p-S6 levels are increased in the sleep restricted animals (Figure 10).

**Table 4:** Repeated measures ANOVA results for pS6 expression.

Interaction	Main effect	*F*_(df,error)_ value	*P*-value
Region × condition × pS6		*F*_(7,36)_ = 0.345	0.920
Condition × pS6		*F*_(2,11)_ = 0.013	0.987
Region × pS6		*F*_(3,36)_ = 0.282	0.855
Region × condition		*F*_(10,55)_ = 1.238	0.288
	Region	*F*_(5,55)_ = 1.109	0.366
	Condition	*F*_(2,11)_ = 4.485	0.038^∗^
	pS6	*F*_(1,11)_ = 0.007	0.937


## Discussion

The data presented in this manuscript highlight the importance of sleep during brain development in C57BL/6J male mice and indicate that even mild but chronic reductions in sleep lead to long-term behavioral changes. Our paper confirms and extends our previous studies (Sare et al., 2015) which indicated that the behavioral response to sleep-restriction may be modulated by sex. In this paper, we studied only male C57BL/6J mice, and we included a group of stressed, but not sleep-restricted animals to control for stress *per se.* We demonstrate that sleep-restriction, but not stress, results in long-lasting hypoactivity and affects relative levels of p-S6, a downstream marker for mTOR activity. Both sleep-restriction and stress effects lead to impaired preference for social novelty after recovery sleep. Even mild reductions in sleep, if chronically sustained, can lead to long-term behavioral and molecular changes.

To control for the effects of stress, *per se*, we included an additional group of mice which were stressed but not sleep-restricted. In contrast to our previous study in which our “stress” control was handled for 10 min per day during the sleep-restriction period (Sare et al., 2015), in the present study, we added a third group in which mice were stressed by gentle prodding but not sleep-restricted. It is important to note that both the “stress” and sleep restriction groups were moved to another room during the study while the control groups were kept in the housing room which may have had an influence on the mice.

In the present study, in addition to measuring the long-term effects of mild, chronic stress on behavior, we also measured serum corticosterone concentrations during the period of sleep-restriction as an index of a stress response. Neither mild chronic stress nor sleep-restriction elevated corticosterone levels in C57BL/6J mice. Unexpectedly, mean corticosterone levels were lower in the stress and sleep-restriction groups compared with control animals at P42. Whereas these effects were not statistically significant, we were concerned that the stressed and sleep-restricted mice had been handled daily during the sleep-restriction period, whereas control mice were not. They may have become accustomed to handling and were, therefore, less stressed during the serum collection procedure than controls. We tested this idea by handling adult control mice daily for 2 weeks prior to serum collection, but results (data not shown) did not support this idea. It is possible that stress and sleep-restriction early in life alter the corticosterone response later in life. This is congruent with studies that show early life handling can alter the stress response and reduce stress to subsequent handling (Korosi and Baram, 2010).

Other studies have shown that there is a long-term effect on behavior following neonatal REM sleep deprivation. In particular, REM sleep deprivation in neonatal rats leads to depressive-like behaviors in adult-hood (Mirmiran et al., 1983; Feng et al., 2001; Feng and Ma, 2003). Rather than total REM sleep deprivation, we focus on the long-term effects of chronic sleep restriction (regardless of sleep stage). In the present study of a new and larger cohort of only male C57BL/6J mice, we extend our previous report (Sare et al., 2015) that chronic mild sleep-restriction during brain development has long-lasting effects on behavior. The most striking behavioral change was hypoactivity in the open field. This effect on behavior was seen both during the sleep-restriction and after 32 days of recovery. Although these results are consistent with our previous results (Sare et al., 2015), they contrast other studies that found hyperactivity in rats following REM sleep deprivation (Mirmiran et al., 1983). The differences between our results and those finding hyperactivity might be a species difference or a difference in the response to either total or REM sleep deprivation.

Other results in the open field arena pertain to anxiety-like behavior or the center to total distance moved. These results are less robust than the hypoactivity finding, but they suggest that sleep-restricted mice do not show the habituation observed in the control and stress groups. This was particularly evident at P84 following recovery and suggests that anxiety-like behavior is enhanced. Our results are in accord with another study which employed chronic REM sleep restriction in young rats followed by a brief recovery period. They found increased anxiety-like behaviors, measured by the elevated plus maze (da Silva Rocha-Lopes et al., 2018). Another possible interpretation of this response is that this second exposure to the open-field arena would be expected to result in more rapid habituation. A lack of habituation might reflect a lack of recognition of the arena in the sleep-restricted mice.

Our results on the test of social behavior, in agreement with our previous results in male mice (Sare et al., 2015), indicate that sleep-restricted males show a deficit in preference for social novelty after recovery sleep. This behavioral test in particular is thought to reflect autistic-like behavior in mice (Silverman et al., 2010). Interestingly, the stress group had a similar behavioral impairment suggesting early life stress may be sufficient to induce social behavior impairments, whereas other behavior impairments appeared to be specific for the loss of sleep.

In this study, we considered several molecular level changes that might underlie or at least be associated with these behavioral effects. There are many factors known to be influenced by acute sleep deprivation that are also implicated in brain plasticity (Picchioni et al., 2014). For example, myelination can be altered by sleep deprivation and myelin formation is associated with developmental plasticity (Picchioni et al., 2014). Whereas we did not find any effects of sleep-restriction on MBP in lysates prepared from frontal cortex, this does not rule out the possibility of localized changes in myelin. Similarly, we did not find effect of sleep-restriction on microglia as indicated by changes in Iba1, but this does not rule out the possibility microglia may have been activated by the sleep-restriction. Such changes might be detected via immunohistochemistry. We also assayed cellular stress through activation of JNK. However, there are other forms of cellular stress (like ER stress and oxidative stress) (Picchioni et al., 2014) that may still be altered following chronic sleep-restriction.

Finally, we examined pathways involved in transcription and translation, which can be altered following sleep-deprivation and have influential roles in the potential pathology of neurodevelopmental disorders (Picchioni et al., 2014). We found that, even after recovery, sleep-restricted animals had increased levels of p-S6 suggesting increased mTORC1 activity. Dysregulation of the mTOR pathway is hypothesized to be a common abnormality in autism (Winden et al., 2018), and mouse models with upregulated p-S6 have been shown to have autistic-like behaviors, including social behavior abnormalities (Reith et al., 2012; Tsai et al., 2012).

These data highlight the importance of sufficient sleep during development for normal behavior. Based on these results, sleep disturbance during development can have a long-lasting influence on behavior, particularly activity and social behavior. Our data support an involvement of the mTORC1 pathway in the behavioral changes. Moreover, our results have implications for our understanding of ASDs and may offer an avenue for pursuing potential treatments.

## Ethics Statement

All procedures were performed in accordance with the National Institutes of Health Guidelines on the Care and Use of Animals and approved by the National Institute of Mental Health Animal Care and Use Committee.

## Author Contributions

RS contributed to the design of the study. RS, AS, ML, AL, IL, CAS, CH, AM, and SC conducted experiments. RS and CBS analyzed data and wrote the manuscript. All authors approved the final version of the manuscript.

## Conflict of Interest Statement

The authors declare that the research was conducted in the absence of any commercial or financial relationships that could be construed as a potential conflict of interest.

## Supplementary Material

FIGURE S1 Social behavior data showing time in all three chambers at P48 **(A,C)** and at P90 **(B,D)** after recovery. This data is a re-representation of Figure 7A,B, 8A,B also showing the time spent in the center chamber for **(A,B)** sociability and **(C,D)** social novelty. Mice traveled in all three chambers. Ear bar is the mean ± SEM for the number of mice indicated in parentheses.

## Abbreviations

AMPK: 5′ AMP-activated protein
ASD: autism spectrum disorders
MBP: myelin basic protein
mTOR: mammalian target of rapamycin
S6: ribosomal protein S6
SEM: standard error of the mean
