# Supplementary figures and images for: Chronic Sleep Restriction in Developing Male Mice Results in Long Lasting Behavior Impairments
Source: Front Behav Neurosci. 2019 May 3;13:90. doi: 10.3389/fnbeh.2019.00090 (PMC6509425; doi:10.3389/fnbeh.2019.00090)

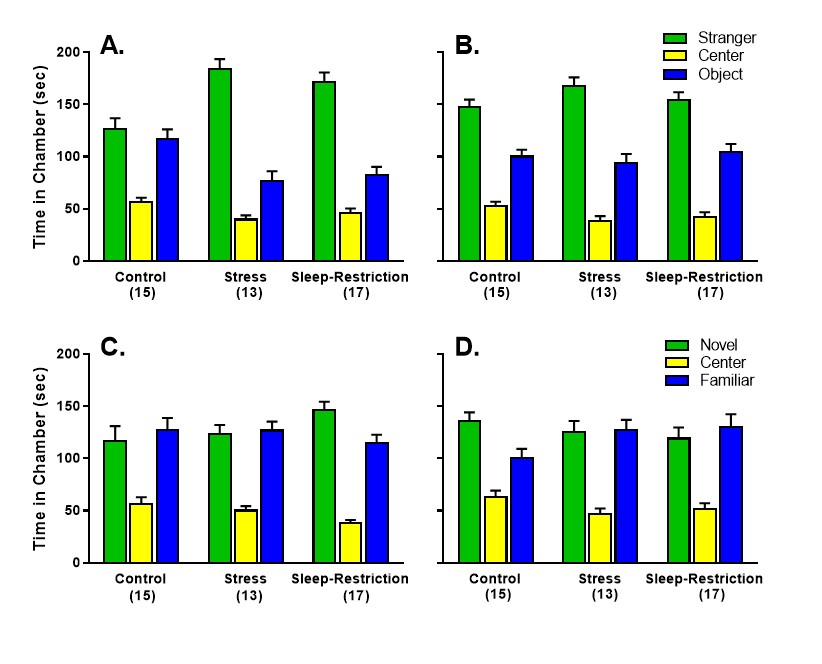

Supplement: FIGURE S1 — Social behavior data showing time in all three chambers at P48 (A,C) and at P90 (B,D) after recovery. This data is a re-representation of Figure 7A,B, 8A,B also showing the time spent in the center chamber for (A,B) sociability and (C,D) social novelty. Mice traveled in all three chambers. Ear bar is the mean ± SEM for the number of mice indicated in parentheses. [file Image_1.JPEG]
